# Association of increased primary breast tumor *AGR2* with decreased disease-specific survival

**DOI:** 10.18632/oncotarget.25225

**Published:** 2018-05-01

**Authors:** Phoebe Ann, Brandon-Luke L. Seagle, Arunima Shilpi, Manoj Kandpal, Shohreh Shahabi

**Affiliations:** ^1^ Division of Gynecologic Oncology, Department of Obstetrics and Gynecology, Northwestern University Feinberg School of Medicine, 60611 Chicago, IL, USA

**Keywords:** AGR2, ER+ breast cancer, primary tumor mRNA

## Abstract

**Objective:**

Tumor expression of Anterior Gradient 2 (*AGR2*), an endoplasmic reticulum protein disulfide isomerase, was associated with decreased breast cancer survival. We aimed to validate the association of tumor *AGR2* mRNA expression with disease-specific survival (DSS) and identify differentially expressed signaling pathways between high and low *AGR2* expression tumor groups.

**Methods:**

Primary tumor mRNA expression data from the METABRIC study was used to evaluate *AGR2* expression as a prognostic factor for DSS while adjusting for survival-determining confounders using Cox proportional-hazards regression. Differentially expressed genes and signaling pathway differences between high and low *AGR2* groups were determined by modular enrichment analyses using DAVID and Ingenuity Pathway Analysis.

**Results:**

Increased tumor *AGR2* mRNA expression was associated with decreased DSS among 1,341 women (per each standard deviation increase of *AGR2* expression: HR 1.14, 95% CI: 1.01-1.29, P = 0.03). Pathway analyses supported prior experimental studies showing that estrogen receptor 1 (*ESR1*) regulated *AGR2* expression. Canonical signaling pathways significantly differentially represented between high and low *AGR2* groups included those involved in inflammation and immunity.

**Conclusion:**

Increased primary tumor *AGR2* expression was associated with decreased DSS. Pathway analyses suggested that increased *AGR2* was associated with endoplasmic reticular homeostasis, possibly allowing tumor cells to overcome hypoxic stress and meet the increased protein demand of tumorigenesis, thereby preventing unfolded protein response-mediated apoptosis.

## INTRODUCTION

Breast cancer is the leading cause of cancer-related deaths among women worldwide [1]. Over 1.7 million women are diagnosed with breast-cancer annually [1]. Despite efforts at early detection, 30-40% of women are diagnosed with metastatic cancer and die from therapy-resistant disease [2]. Over 70% of breast cancers are estrogen receptor positive (ER+), with well-known estrogen-driven malignant transformation and therapy resistance [3–5]. Anterior Gradient 2 (AGR2) is a protein disulfide isomerase in the endoplasmic reticulum first discovered in ER+ breast cancer cells [6–8]. AGR2 inhibits the tumor suppressor p53, promotes cell survival and proliferation, and mediates metastatic spread in breast cancer cells [9–12]. *AGR2* expression is associated with decreased survival among women with ER+ breast cancer as well as tamoxifen and fulvestrant resistance [12–14].

A substantial body of recent experimental research has shown that estrogen-mediated activation of estrogen receptor directly targets *AGR2* for active gene transcription. To begin with, AGR2 and ESR1 protein expression has been shown by immunohistochemistry to be positively correlated in both ER+ breast cancer cell lines and ER+ breast tumors [8, 11, 15, 16]. *AGR2* knockdown by siRNA, shRNA, and miRNA in ER+ breast cancer lines reduces growth, survival, and migration, as well as fulvestrant and tamoxifen resistance [12, 17–19]. Estradiol treatment of ER+ breast cancer cell lines stimulates both AGR2 expression and a twofold increase in ESR1 binding to the *AGR2* promoter region as detected by chromatin immunoprecipitation (ChIP) [12, 20]. ESR1 was shown to increase *AGR2* expression from a transiently transfected *AGR2* promoter reporter plasmid [12]. ChIP-Seq and chromatin interaction analysis by paired-end tag sequencing (ChIA-Pet) studies defining global ER-binding sites further support the targeted binding and transcriptional activation of *AGR2* by ER in cell lines and primary tumor tissue, and an increased number of occupied ER-binding sites may correlate with poor prognosis [14, 21–23]. Altogether, these studies provide strong experimental evidence that estrogen-mediated activation of ESR1 directly upregulates *AGR2* gene transcription.

Two studies have reported an association of increased protein-level *AGR2* expression and decreased breast cancer survival using tumor immunohistochemistry. One analysis is adjusted for some clinical variables, and the other is not confounder-adjusted [12, 24]. A third retrospective study of *AGR2* as a predictor of disease-free survival reports a significant association of decreased survival and increased tumor *AGR2* mRNA expression using qRT-PCR among 78 women with tamoxifen-treated ER+ breast cancer but without adjustment for potential confounders [16].

Now, large breast cancer cohorts with clinical follow-up and multiplatform –omics primary tumor data have been reported. These studies expand our understanding of molecular subtypes of breast cancer as well as genetic prognostic factors, such as specific tumor mutations, in breast cancer [25–27]. These large -omics datasets utilize the gold standard design, the prospective observational cohort study, for the discovery and validation of disease prognostic factors. Therefore, they allow us to confirm and further explore previously experimentally demonstrated gene associations with actual patient data, not only with breast tumor gene expression but also with investigation of other clinical variables such as stage, tumor size, and lymph node metastasis. Here, primary tumor mRNA data from women in the Molecular Taxonomy of Breast Cancer International Consortium (METABRIC) breast cancer cohort were analyzed for *AGR2* expression and disease-specific survival. Differentially expressed genes and their cellular pathways between women with tumors having either high and low *AGR2* expression were also explored.

## RESULTS

### Validation of the *AGR2* survival association

Clinical and primary tumor mRNA expression data from 2,000 fresh-frozen breast cancer specimens from the METABRIC study were analyzed for prognostic gene associations. Cohort selection for analysis excluded tumor specimens with benign or rare histological types or missing clinical data; Table 1 shows baseline characteristics of the cohort. Single-gene and multigene survival analyses identified *AGR2* and Estrogen Receptor 1 (*ESR1*) as significantly associated with DSS in highly significant Cox proportional-hazards regression models (Table 2). Each one standard deviation increase in relative expression of *AGR2* was associated with 14% increased hazard of death from disease (HR 1.14 (1.01-1.29), P = 0.03). Conversely, each one standard deviation increase in relative expression of *ESR1* was associated with 18% decreased hazard of death from disease (HR 0.82 (0.69-0.99), P = 0.04). Increased *AGR2* mRNA expression was correlated with increased *ESR1* mRNA expression (Spearman’s rho = 0.547, P < 0.001).

**Table 1:** Patient characteristics of METABRIC sample for Disease-specific survival multivariate model

Patient characteristic	Patient sub-characteristic	n = 1341
Disease-specific survival (months)		117.60 [62.33, 188.73]
Age at diagnosis (years)		61.12 [50.92, 69.83]
Tumor stage (%)	1	455 (33.9)
	2	769 (57.3)
	3	110 (8.2)
	4	7 (0.5)
Lymph Node Positivity Status (%)	Negative	714 (53.2)
	Positive	627 (46.8)
Tumor size (cm)		2.20 [1.70, 3.00]
ER Positivity Status (%)	Negative	298 (22.2)
	Positive	1043 (77.8)
HER2 Positivity Status (%)	Negative	1176 (87.7)
	Positive	165 (12.3)
Breast surgery (%)	Breast-conserving	574 (42.8)
	Mastectomy	767 (57.2)

Survival model n = 1341. Results for Disease-specific survival, Age at diagnosis, and Tumor size reported as “median [interquartile range].” All others reported as “absolute value (percentage).”

**Table 2:** Hazards of death from single and multivariate Cox regression of disease-specific survival

Affymetrix U133A microarray mRNA expression dataset	HR (95% CI), *P*
	DSS (n = 1341) Deaths = 455
**Multivariate model**	
*AGR2*	1.14 (1.01-1.29), 0.03
*ESR1*	0.82 (0.69-0.99), 0.04
Age_per year_	1.01 (1.00-1.02), 0.004
Mastectomy, compared to breast-conserving surgery	1.31 (1.07-1.61), 0.01
Lymph node metastasis	1.73 (1.34-2.24), <0.001
Tumor Size_per cm_	1.12 (1.06-1.19), <0.001
GATA3 mutation	0.67 (0.47-0.96), <0.03
TP53 mutation	1.55 (1.23-1.95), <0.001
Stratification variables	ER IHC status, HER2 expression status, stage
Model *P*	< 1.3 x 10^-14^

HR (95% CI), P: Hazard ratio (95% confidence interval) and p-value, scaled to one standard deviation of gene expression for *AGR2* and *ESR1*; DSS: Disease-specific survival.

**Figure 1:**
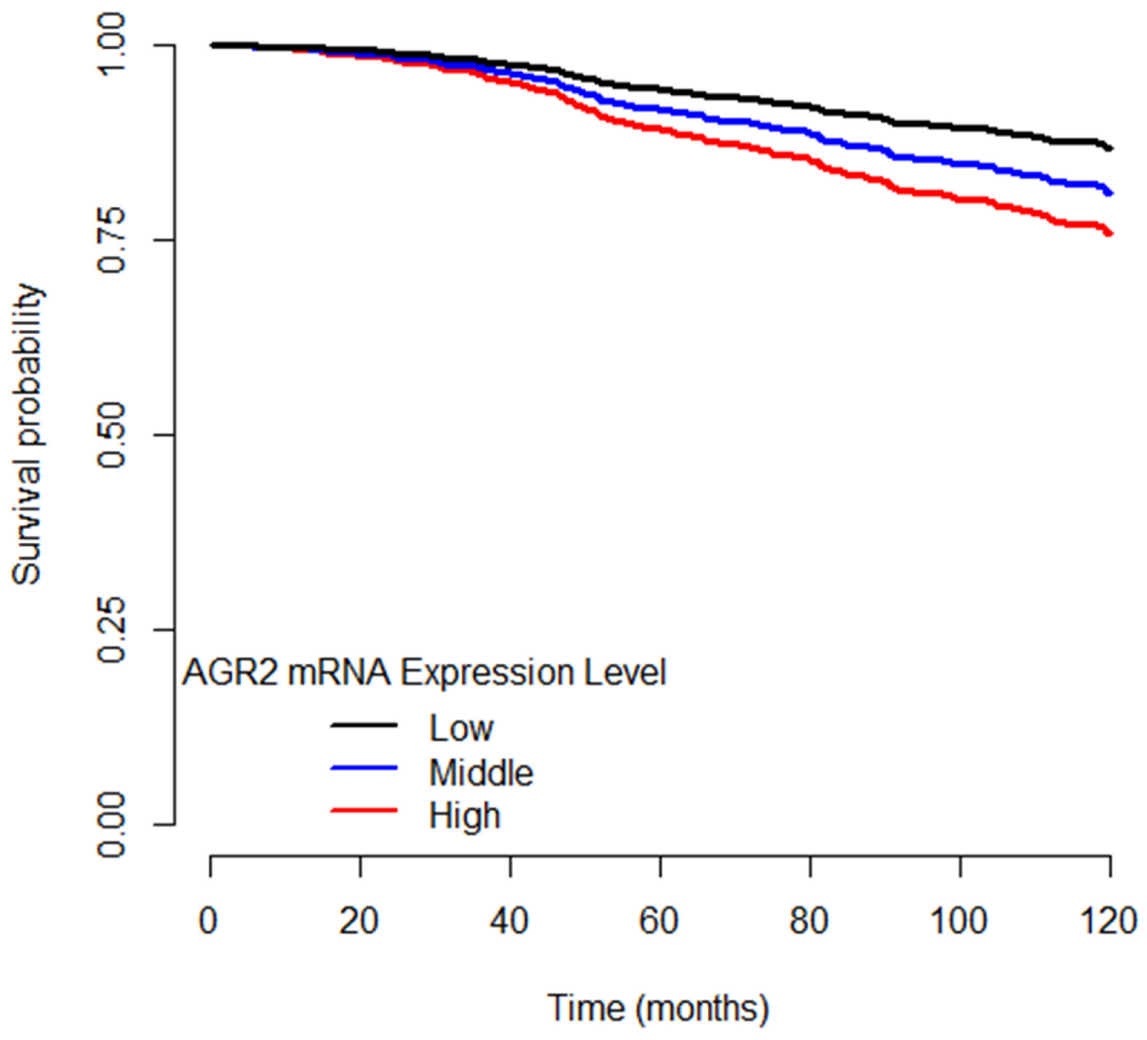
Disease-specific survival curves of ER+ breast cancer patients separated by *AGR2* mRNA expression level High AGR2 mRNA expression level is designated as z ≥ 1.5. Low AGR2 mRNA expression level is designated as z ≤ -1.5. Middle AGR2 mRNA expression level is designated as -1.5 < z < 1.5.

Clinical and molecular covariates were included in the single and multigene Cox proportional-hazards models to adjust for potential confounders of DSS. Clinical covariates significantly associated with DSS were age, breast surgery (breast-conserving vs. mastectomy), presence of positive lymph nodes, and tumor size. Molecular covariates significantly associated with DSS were *AGR2* and *ESR1* mRNA expression levels, and *GATA3* and *TP53* mutation statuses, both tumor mutations with known survival associations (Table 2). Table 3 shows predicted five-year survival differences comparing women with high (z > 1.5) versus low (z < -1.5) *AGR2* tumor mRNA expression while modeling various stages and lymph node status combinations from the Cox regression. Figure 1 shows characteristic, predicted disease-specific survival curves.

**Table 3:** Disease-specific survival (DSS) rates of ER+/HER2- mastectomy patients with high or low primary breast tumor AGR2 mRNA microarray expression (METABRIC)

Tumor Stage	Tumor Size (cm)	Lymph Node Positivity Status	AGR2 mRNA expression	DSS at 60 mo (%)	Difference in DSS at 60 mo between high and low AGR2 (%)	DSS at 120 mo (%)	Difference in DSS at 60 mo between high and low AGR2 (%)
I	1.7	Negative	High	91.63%	-3.99%	77.66%	-10.19%
			Low	95.62%		87.85%	
	1.7	Positive	High	85.94%	-6.59%	64.50%	-15.37%
			Low	92.53%		79.87%	
II	2.2	Negative	High	89.15%	-5.13%	75.86%	-10.94%
			Low	94.28%		86.80%	
	2.5	Negative	High	88.76%	-5.31%	75.08%	-11.26%
			Low	94.07%		86.34%	
	2.5	Positive	High	81.32%	-8.62%	60.83%	-16.68%
			Low	89.95%		77.51%	
III	4.5	Positive	High	72.75%	-12.20%	52.94%	-19.24%
			Low	84.96%		72.18%	
IV	3.1	Negative	High	24.80%	-24.14%	24.80%	-24.14%
			Low	48.94%		48.94%	
	3.1	Positive	High	8.91%	-20.04%	8.91%	-20.04%
			Low	28.95%		28.95%	

Disease-specific survival rates of ER+/HER2- mastectomy patients were calculated with ESR1 mRNA microarray expression set to median 10.7, and age at diagnosis set to median 61 years. AGR2 mRNA expression levels “High” are those for which z-score > 1.5, and “Low” are those for which z-score < -1.5.

Tumor size for each respective stage represents the median tumor size for patients with that stage. The DSS rates calculated for Stage II and tumor size = 2.2 cm represent that with the most common stage and the median tumor size across all stages.

### Identification of DEGs and major molecular pathways by *AGR2* prognostic groups

Relative hazard of disease-specific death by *AGR2* expression suggested z-score values of ± 1.5 as cut-point values to define *AGR2* prognostic groups as high and low *AGR2* expression breast tumor groups. The Benjamini-Hochberg (BH) q-value is a widely accepted, stringent adjustment of the standard p-value to decrease the false discovery rate (FDR) in the circumstance of multiple comparisons [28, 29], and we have used multiple BH q-value cutoffs to most effectively minimize the false discovery rate. Differentially expressed genes (DEGs) between these high (mRNA expression z-score levels of *AGR2* > 1.5) and low (z-score < -1.5) *AGR2* expression tumor groups from the METABRIC study were identified using the cutoff of BH q-value < 0.05. To verify our results from the METABRIC study, we also identified DEGs from RNA sequencing data from tumor mRNA expression from the TCGA study of human breast tumors with BH q-value < 0.05. Gene enrichment studies provided lists of more than 5,000 DEGs from each cohort, METABRIC and TCGA. We performed modular enrichment analysis of the 3,000 most statistically significant DEGs in each cohort; significantly enriched KEGG pathways are shown in Table 4. We used a second cutoff, the log_2_ of gene expression fold-change of 1.5 to generate a subset of the DEGs that are most differentially expressed from each cohort (resulting in 790 genes from METABRIC, and 1143 genes from TCGA) for further pathway analyses by Ingenuity Pathway Analysis (IPA). IPA of the DEGs from each cohort yielded significantly overrepresented pathways and functional networks between high and low *AGR2* groups (Table 5), with cutoffs for pathway significance set to BH q-values < 0.050 and <0.200 (Supplementary Table 1). Although, IPA does not recommend a strict cutoff, q-value < 0.250 is an accepted standard for gene set analysis tools like DAVID and gene set enrichment analysis [30, 31].

**Table 4:** KEGG pathways over-represented in the high AGR2 group (z > 1.5) vs. the low AGR2 group (z < -15)

Breast Cancer Study	Enriched KEGG pathways	Benjamini-Hochberg q-value, regulation change^a^
METABRIC	Pathogenic Escherichia coli infection	0.01, down
	Cell cycle	0.01, down
	HTLV-I infection	0.01, down
	TNF signaling pathway	0.03, down
	NF-kappa B signaling pathway	0.03, down
TCGA	Cell cycle	0.05, down

Regulation change: Listed as up or down if ≥ 2/3^rd^ genes in cluster share the same up or down directional expression change.

**Table 5:** Top IPA pathways over-represented in the high AGR2 group (z > 1.5) vs. low AGR2 group (z < -15)

Breast Cancer Study	Enriched IPA canonical pathways	Benjamini Hochberg q-value^a^, directional regulation change^b^
METABRIC	Estrogen-mediated S-phase Entry	0.005, down
	Cyclins and Cell Cycle Regulation	0.005, mixed
	Glioblastoma Multiforme Signaling	0.010, down
	Cell Cycle: G1/S Checkpoint Regulation	0.010, down
	Glioma Signaling	0.012, down
TCGA	Wnt/Beta-catenin Signaling	0.079, mixed
	Estrogen-mediated S-phase Entry	0.199, down
	Antiproliferative Role of TOB in T Cell Signaling	0.199, down
	Cyclins and Cell Cycle Regulation	0.199, mixed
	Anandamide Degradation	0.199, up

^a^Cutoffs for pathway significance were set to BH q-values < 0.050 and < 0.200. The full list of significant pathways for METABRIC and TCGA can be found in Supplementary Table 1.

^b^Regulation change: Listed as up or down if ≥ 2/3^rd^ genes in cluster share the same up or down directional expression change, otherwise listed as mixed if general directional change is unclear.

We also performed permutation analysis to verify that these IPA pathways were dependent on the specific list of genes and not enriched by chance. Supplementary Table 2 shows the number of pathways enriched to q-value < 0.050 and < 0.200, and Supplementary Table 3 shows the number of times that the original significantly enriched METABRIC IPA pathways (Table 5) were significantly enriched in IPA with random permutation of gene names to fixed columns of gene expression fold change. Importantly, the majority of the top IPA pathways originally significantly enriched in METABRIC and TCGA were not found to be significantly enriched in permutation analyses (Supplementary Table 3, Supplementary Table 4). Specifically, IPA pathways with direct roles in breast cancer pathophysiology such as Estrogen-mediated S-phase Entry, Cyclins and Cell Cycle Regulation, and Cell Cycle: G1/S Checkpoint Regulation were not significantly enriched in permutation analyses. Our results strongly support that the METABRIC and TCGA significantly enriched IPA pathways were not enriched by chance and were dependent on the list of differentially expressed genes between high and low *AGR2* expression groups.

## DISCUSSION

We validated that *AGR2* and *ESR1* mRNA expression levels are significantly associated with DSS (*AGR2:* HR 1.14 (1.01-1.29), P = 0.03; *ESR1*: HR 0.82 (0.69-0.99), P = 0.04), and that *AGR2* and *ESR1* mRNA expression were correlated. We then determined differentially expressed genes between tumors grouped by high or low *AGR2* mRNA expression and retrieved significantly enriched KEGG and IPA pathways, which represented those involved in cell cycle progression, inflammation, and immune response.

*AGR2* is an endoplasmic reticulum-resident protein disulfide isomerase with increased expression levels that are implicated in several cancers, including breast, ovarian, prostate, bladder, and pancreatic cancer [8, 11, 14–16, 32–37]. It is hypothesized that increasing *AGR2* within the endoplasmic reticulum allows cancer cells to adapt to higher secretory protein synthesis demands during tumorigenesis and metastasis [6]. For example, *AGR2* is overexpressed and secreted by both bladder and breast cancer cells [36, 38]. Intracellular versus secreted *AGR2* had different roles in enhancing breast cancer fulvestrant resistance [18]. These studies provided mechanistic biological plausibility to a meaningful role of *AGR2* in metastasis and hormone therapy resistance.

We validated the negative correlation between *AGR2* primary tumor mRNA expression levels and disease-specific survival using data from a larger cohort of women with available information for confounder-adjustment by a variety of potential clinical and molecular confounders. *AGR2* expression was inversely associated with DSS, while *ESR1* expression was directly associated with DSS. Since *ESR1* signaling increases *AGR2* expression but estrogen receptor expression itself is associated with increased survival, it may be difficult to observe the independent negative prognostic association of *AGR2* with decreased survival. Furthermore, because the expression of these two genes is correlated, and collinearity tends to decrease observed statistical significance in regression models, this may explain why the observed p-values are not lower even in this relatively large cohort. Predictably, increased *ESR1* expression was associated with increased DSS, consistent with ER+ breast cancers being more differentiated, less aggressive, and often responsive to hormone therapy [39, 40]. The positive Spearman’s correlation between *AGR2* and *ESR1* agreed with prior literature showing that *AGR2* expression is directly stimulated by estrogen signaling [14]. The opposing associations of *AGR2* and *ESR1* expression with DSS could reflect the role of *AGR2* in hormone therapy- resistant ER+ breast cancer: ER+ breast cancers may develop resistance by overexpressing *AGR2* [12–14]. Indeed, the downregulation of the “Estrogen-mediated S phase entry” pathway in breast tumors with high *AGR2* mRNA expression—suggested by IPA analyses in both the METABRIC and TCGA datasets—may represent breast tumors that overexpress *AGR2* to become resistant to estrogen therapy (Table 5). Furthermore, while *ESR1* can initially upregulate *AGR2* expression, breast cancer cells that achieve tamoxifen resistance no longer require *ESR1* for *AGR2* expression, likely due to an alteration in the activity of the transcription factor *FOXA1* [19]. A combined strategy to block *AGR2* expression or function in combination with anti-estrogenic hormonal therapy may be a novel strategy for the treatment of ER+ breast cancers.

mRNA expression fold changes in DEGs of breast tumors with high *AGR2* compared to those with low *AGR2* were consistent with prior findings highlighting the roles of intracellular and extracellular AGR2 in tumorigenesis pathways mediated by estrogen signaling and Insulin Growth Factor 1 (IGF-1) [38]. Indeed, *IGF1* receptor (*IGF1R*) was significantly upregulated in tumors with high *AGR2* mRNA expression compared to those with low *AGR2* mRNA expression (METABRIC microarray *IGF1R* mRNA fold change = 3.03, TCGA RNA Seq *IGF1R* mRNA fold change = 3.13). Increased *AGR2* mRNA expression was correlated with increased *IGF1R* mRNA expression (Spearman’s rho = 0.451, p < 0.001). Recent studies have shown that particular *IGF1R* polymorphisms significantly increase the risk of early tumor progression in tamoxifen-treated ER+ breast cancer, and that inhibition of IGF1R activity enhances response to trastuzumab therapy [41, 42]. Therefore, IGF1R-mediated signaling likely plays a role in *AGR2*-mediated trastuzumab and tamoxifen resistance in ER+ breast cancer tumors and is worthy of further investigation.

The association of increased *AGR2* mRNA expression and decreased disease-specific survival may also be because higher expression of the endoplasmic reticulum-resident protein disulfide isomerase *AGR2* allows for homeostatic adaptation to an increased demand on protein synthesis and secretion in oncogenesis. Tumor cells are challenged by hypoxia, nutrient deficiency, and increased proteomic demand. If the tumor cell cannot counteract these pathological conditions successfully, proper protein folding and endoplasmic reticulum homeostasis will be disturbed, ultimately causing endoplasmic reticulum stress. Endoplasmic reticulum stress activates the unfolded protein response (UPR), which is tailored to re-establish endoplasmic reticulum homeostasis by shutting down certain types of protein translation, upregulating endoplasmic reticulum folding machinery components, and boosting endoplasmic reticulum quality control mechanisms such as endoplasmic reticulum-associated degradation [43]. *AGR2* expression is modulated by endoplasmic reticulum stress-induced UPR and in turn maintains endoplasmic reticulum homeostasis. Basal levels of intracellular AGR2 are controlled by IRE1-alpha and ATF6-alpha, two of the three key arms of the UPR [44]. In the gastrointestinal tract, AGR2 is critically required for Mucin 2 production, and in its absence, the intestinal epithelium exhibits increased endoplasmic reticulum stress markers such as Grp78 and Xbp1 splicing, as well as elevated pro-inflammatory cytokines [45–47]. Consequently, *AGR2* knockout mice spontaneously develop severe ileocolitis that histopathologically resembles human Crohn’s disease [47]. Furthermore, endoplasmic reticulum stress induces *AGR2* expression in inflammatory pre-neoplastic pancreatic tissue. By enhancing endoplasmic reticulum folding capacity, AGR2 allows pre-cancerous cells to accommodate increased protein demand both before and after oncogenic mutations such as that of Kras, ultimately leading to pancreatic cancer progression [48]. In normal breast development, *AGR2* is maximally expressed during late pregnancy and lactation to accommodate the increased demand on protein synthesis and secretion during these specific physiological conditions [20]. It is therefore plausible that AGR2 is involved in the UPR to resolve endoplasmic reticulum stress, and that breast cancer cells can utilize the homeostatic effects of *AGR2* to overcome their own pathologically elevated need for protein synthesis and secretion. When endoplasmic reticulum stress is too severe, the UPR turns from a pro-survival to a pro-death response [49]. Pro-death UPR signals, including those mediated by CHOP, GADD34, ERO1-alpha, and Bcl-2, tip the balance towards apoptosis as well as activate inflammatory signaling cascades [50–53]. It is possible that breast cancer cells that fail to upregulate *AGR2* likewise fail to mount a successful pro-survival UPR that restores endoplasmic reticulum homeostasis, thus succumbing to pro-death UPR signals that promote apoptosis and inflammation. UPR-induced apoptosis includes apoptosis mediated by the transcription factor CHOP, which can promote the transcription of pro-apoptotic BH3-only proteins such as Noxa and Puma through p53-dependent mechanisms [54, 55]. Since AGR2 has been shown to inhibit p53 activation through a DUSP10-mediated pathway, it is likely that high AGR2 levels relieve endoplasmic reticulum stress and thus stave off pro-death UPR-mediated apoptosis [10].

Additionally, pro-death UPR signals activate classical inflammatory signaling cascades, including the production of pro-inflammatory molecules like IL-6, IL-8, and TNF-alpha, as well as acute-phase response neutrophilia and associated production of cytokines like IL-1beta and IL-2R [53, 56–58]. NF-kB carries out pro-death UPR-mediated inflammation, and it is a known activator of p53 [56, 59, 60]. The KEGG NF-kB signaling pathway is downregulated in breast tumors with high *AGR2* expression compared to those with low *AGR2* expression within the METABRIC microarray dataset (Table 4). Furthermore, this NF-kB pathway downregulation, and the fact that *AGR2* can inhibit p53 activity, suggest that *AGR2* prevents both pro-death UPR-mediated apoptosis and inflammation, ultimately promoting tumor cell survival and proliferation.

UPR-induced inflammation can be critical for anti-tumor immunity, since activated leukocytes can present tumor antigens to CD4+ T cells [61, 62]. Consequently, tumor-infiltrating lymphocytes have been shown to be favorable for breast cancer survival, and UPR activation is associated with tumor-infiltrating lymphocytes in breast cancer [63, 64]. In both the METABRIC and TCGA datasets, downregulation of genes such as *CD4* and *CD40* may indicate decreased CD4+ T cell response or decreased *CD40*-mediated apoptosis, both of which are correlated with tumor regression, better response to therapy, and higher overall breast cancer survival [65, 66]. Furthermore, breast tumors that overexpress *AGR2* exhibit downregulation of KEGG pathways epitomized by the inflammatory response, including Pathogenic Escherichia Coli infection, HTLV-1 infection, and TNF pathway (Table 4). UPR activation in immune cells and various stromal cells can induce TNF-alpha secretion, and conversely TNF-alpha can trigger UPR activation in the liver to amplify the inflammatory response [53, 58, 67, 68]. The close interplay between the UPR and the TNF pathway, plus recent findings that show that TNF-alpha can prevent *in vivo* breast cancer development, suggest that downregulation of the TNF pathway in *AGR2*-overexpressing breast cancers may contribute to their hormone therapy resistance [69].

The downregulation of mRNA expression of cell cycle pathway genes in tumors with higher AGR2 expression may be due to preservation technique of the NCDB database tumor samples, a technique which may under-represent extracellular AGR2, whose function has been proven to be distinct from that of intracellular AGR2 protein. While extracellular AGR2 promotes cell cycle progression, intracellular AGR2 functionally interacts with ER [18]. Furthermore, AGR2 has been shown to promote cell cycle progression via induction of cell cycle proteins such as cyclin D1, and extracellular AGR2 knockdown using anti-AGR2 antibodies in three ER+ breast cancer cell lines significantly reduces cyclin D1 protein levels and cell growth [17]. Thus, downregulation of cell cycle pathway genes in high AGR2 mRNA expression tumors may reflect the differing roles of extracellular and intracellular AGR2 and the under-measurement of extracellular AGR2 in our analyses.

Limitations of our study include the inherent possibility of causal inference and the risks of selection biases and unmeasured confounding that are relevant to observational cohort studies, with consequent concerns for generalizability, although we used the largest breast cancer study with available –omics data, adequate clinical follow-up, and a disease-specific survival outcome. The TCGA breast cancer cohort was not useful for a validation of the *AGR2* survival association due to inadequately short follow-up time of the cohort. With a large sample size of n = 1,341, multivariable adjustment for several known clinical and molecular confounders, and consistency of our results with prior literature, we believe that the survival association is unlikely to represent a false-positive signal. Given consistency with prior studies using a better powered and more rigorous analysis, we see these results as an independent cohort validation of the previously reported *AGR2* survival association. Another limitation is that the overall dataset used has missing data for some cases. Finally, the discussion above highlights the significant body of laboratory research that has established the biological plausibility for *AGR2* expression as a prognostic biomarker and potential therapeutic target in breast cancer, lending credence to our findings.

In conclusion, we validated the *AGR2* survival association in breast cancer and explored various pathways through which AGR2 mediates poor response to treatment and thus decreased disease-specific survival. These pathways involve *ESR1*, *IGF1R*, and pro-death UPR-mediated apoptosis and inflammation with subsequent recruitment of tumor-infiltrating lymphocytes. As therapy inefficacy and subsequent metastases drive breast cancer mortality, further elucidation of both intracellular and extracellular AGR2 biology and its ability to confer hormone therapy resistance may lead to improved understanding of breast cancer mortality. Simultaneous suppression of AGR2 and ESR1 activity represents a potentially promising concept to be further investigated for breast cancer.

## MATERIALS AND METHODS

### Data source

We performed an observational retrospective cohort analysis of women with breast cancer with publicly available clinical and primary tumor mRNA expression and mutation data from the METABRIC study. Data was retrieved using the cBioPortal implementation in R, package CGDS-R [25, 26, 70]. The METABRIC study included over 2,000 fresh-frozen breast cancer specimens and a subset of normal breast tissue from tumor banks in the UK and Canada. The METABRIC study reported 2,136 primary tumors with expression array (Affymetrix U133A microarray) data and 2,433 primary tumors with somatic mutation testing by sequencing for 173 genes. We also analyzed RNA sequencing data for mRNA expression from 1,100 primary breast cancer tumors from The Cancer Genome Atlas (TCGA) to validate our results for differentially expressed genes from the METABRIC study [27].

### Cohort selection

Women with publicly available clinical, mutation, and mRNA expression data were selected (n = 1,889) from the Curtis et. al. METABRIC cohort [26]. Women with intraductal or intralobular histological types were included; those with benign or rare histological types were excluded (new n = 1,854). Women who did not undergo surgery (breast-conserving or mastectomy) were excluded (new n = 1,835). Women without reported disease stage were also excluded (new n = 1,370). Survival analyses were limited to women with no missing data for any potential confounder that was included in Cox model (final n = 1,341).

### Covariate selection and definitions

Initial covariates for survival analysis included age at diagnosis, breast surgery, cellularity, treatment with adjuvant chemotherapy, claudin subtype, ER IHC status, grade, *HER2* expression status, *HER2* copy number alterations, hormone therapy, menopause status, integrative cluster (METABRIC study molecular subtype), laterality, number of positive lymph nodes, Nottingham prognostic index, PR expression status, treatment with adjuvant radiotherapy, tumor size, and tumor stage. Additional specific molecular covariates included the somatic tumor mutation status of genes with known breast cancer survival associations among women in the METABRIC cohort: *TP53*, *GATA3*, *MAP3K1*, *NF1*, *PIK3CA*, *SMAD4*, and *USP9X*. Also included were the mRNA expression levels of genes that regulate *AGR2* or are potentially similar to *AGR2* including *ESR1, ESR2*, and *AGR3* [6, 7, 10, 12, 14, 15, 24, 25, 71–75]. Molecular covariates utilized for final analyses on different subgroups within breast cancer tumors included *ESR1*, *TP53* mutation, and *GATA3* mutation.

### Single and multiple-gene *AGR2* prognostic biomarker evaluation

*AGR2* was evaluated as a potential prognostic biomarker using single and multiple-gene multivariable Cox proportional-hazards models of disease-specific survival (DSS) as functions of continuous mRNA expression (n = 1,341). Clinical and molecular covariates were included to adjust for potential confounders of DSS. The proportional hazards assumption was checked and the regression model was stratified by estrogen receptor (ER) immunohistochemistry (IHC) status, *HER2* expression status, and stage to maintain proportional hazards. Hazard ratios (HR) are reported per each one standard deviation increase in gene expression to aid between gene comparisons of relative effect estimates in terms of change in HR values per each standard deviation increase in mRNA expression. R and the “survival” and “rms” packages were used for statistical computing [76–78].

### Identification of differential gene expression patterns and major molecular pathways by high and low *AGR2* tumor expression groups

First, we used the final Cox model for DSS and modeled the restricted cubic splines of tumor *AGR2* mRNA expression as a continuous variable to verify that the relationship of *AGR2* mRNA expression with DSS is linear. Next, differentially expressed genes (DEGs) between high and low *AGR2*, with high (mRNA expression z-score levels of *AGR2* > 1.5) vs. low (z-score < -1.5), were determined using Linear Models for Microarray with the limma package in R [79, 80]. We further verified our results for differentially expressed genes using RNA sequencing data for tumor mRNA expression from the TCGA study of human breast tumors. Significant differences were deemed those with a Benjamini-Hochberg procedure q-value < 0.05, to account for multiple comparisons. Gene enrichment studies provided lists of more than 5000 DEGs from each cohort, METABRIC and TCGA. Modular enrichment analyses of the most statistically significant (by q-value) 3,000 DEGs were performed using DAVID [30, 81]. Significantly enriched functional annotation clustering and KEGG pathways were determined and ranked to show overrepresented pathways coincident with higher *AGR2* prognostic expression levels [82]. Ingenuity Pathway Analysis (IPA) (Version 01-07) was performed on 790 and 1143 genes, respectively, from the DEG lists obtained from the METABRIC and TCGA datasets, with an BH q-value < 0.001 and a log_2_ fold change cutoff of 1.5. Cutoff for IPA pathway significance was set to BH q-values < 0.050 and <0.200. Permutation analysis of IPA was performed to validate that IPA pathways were not enriched by chance. IPA was run with random permutation of gene names to fixed columns of gene expression fold change. Ensembl and GeneCards were used to gather background information about relevant gene functions and expression [83, 84].

## SUPPLEMENTARY MATERIALS TABLES




