# Supplementary material for: Association of increased primary breast tumor AGR2 with decreased disease-specific survival
Source: Oncotarget. 2018 May 1;9(33):23114–25. doi: 10.18632/oncotarget.25225 (PMC5955412; doi:10.18632/oncotarget.25225)
Supplement: Supplementary file 1 [file oncotarget-09-23114-s001.pdf]

Association of increased primary breast tumor *AGR2* with decreased disease-specific survival

SUPPLEMENTARY MATERIALS

Supplementary Table 1: IPA pathways over-represented (q-value < 0.200) in the high *AGR2* group (*z* > 1.5) vs. low *AGR2* group (*z* < -1.5)

Breast Cancer Study	Enriched IPA canonical pathways	Benjamini Hochberg q-value
**METABRIC**		
	Estrogen-mediated S-phase Entry	0.005
	Cyclins and Cell Cycle Regulation	0.005
	Glioblastoma Multiforme Signaling	0.010
	Cell Cycle: G1/S Checkpoint Regulation	0.010
	Glioma Signaling	0.013
	Aryl Hydrocarbon Receptor Signaling	0.013
	Antiproliferative Role of TOB in T Cell Signaling	0.017
	Bladder Cancer Signaling	0.017
	Estrogen-Dependent Breast Cancer Signaling	0.020
	Wnt/Î²-catenin Signaling	0.020
	Ovarian Cancer Signaling	0.031
	Regulation of Cellular Mechanics by Calpain Protease	0.035
	Anandamide Degradation	0.041
	HER-2 Signaling in Breast Cancer	0.041
	Non-Small Cell Lung Cancer Signaling	0.054
	Glutathione-mediated Detoxification	0.095
	Wnt/Ca+ pathway	0.111
	Role of NANOG in Mammalian Embryonic Stem Cell Pluripotency	0.117
	Prostate Cancer Signaling	0.155
	LPS/IL-1 Mediated Inhibition of RXR Function	0.155
	Salvage Pathways of Pyrimidine Ribonucleotides	0.155
	Cell Cycle Regulation by BTG Family Proteins	0.157
	Colorectal Cancer Metastasis Signaling	0.158
	ErbB Signaling	0.158
	Regulation of the Epithelial-Mesenchymal Transition Pathway	0.158
	Role of Macrophages, Fibroblasts and Endothelial Cells in Rheumatoid Arthritis	0.158
	Superpathway of Inositol Phosphate Compounds	0.162
	Pancreatic Adenocarcinoma Signaling	0.166
	HIPPO signaling	0.188
	Chronic Myeloid Leukemia Signaling	0.191
**TCGA**		
	Wnt/Beta-catenin Signaling	0.079
	Estrogen-mediated S-phase Entry	0.199
	Antiproliferative Role of TOB in T Cell Signaling	0.199
	Cyclins and Cell Cycle Regulation	0.199
	Anandamide Degradation	0.199
	Glutamine Degradation I	0.199

Supplementary Table 2: METABRIC, TCGA, and permutation analyses pathways with q-value < 0.050 and < 0.200

IPA	Pathways with q-value < 0.050	Pathways with q-value < 0.200
METABRIC	14	31
TCGA	0	6
Permuted data 1	0	6
Permuted data 2	0	1
Permuted data 3	0	19
Permuted data 4	1	7
Permuted data 5	1	15
Permuted data 6	4	34
Permuted data 7	2	6
Permuted data 8	1	1
Permuted data 9	1	0
Permuted data 10	0	0

Supplementary Table 3: Number of times METABRIC and TCGA pathways were enriched in permutation analyses (q-value < 0.200)

Breast Cancer Study	Enriched IPA canonical pathways	Number of times pathway was enriched in permutation analyses (q < 0.200)
METABRIC	Estrogen-mediated S-phase Entry	0
	Cyclins and Cell Cycle Regulation	0
	Glioblastoma Multiforme Signaling	2
	Cell Cycle: G1/S Checkpoint Regulation	0
	Glioma Signaling	0
TCGA	Wnt/Beta-catenin Signaling	0
	Estrogen-mediated S-phase Entry	0
	Antiproliferative Role of TOB in T Cell Signaling	1
	Cyclins and Cell Cycle Regulation	0
	Anandamide Degradation	1

Supplementary Table 4: Significantly enriched IPA pathways from permutation analyses (q-value < 0.200).

See Supplementary File 1
