# Supplementary material for: Association of increased primary breast tumor AGR2 with decreased disease-specific survival
Source: Oncotarget. 2018 May 1;9(33):23114–25. doi: 10.18632/oncotarget.25225 (PMC5955412; doi:10.18632/oncotarget.25225)
Supplement: Supplementary file 2 [file oncotarget-09-23114-s002.docx]

Supplementary Table 4. Significantly enriched IPA pathways from permutation analyses (q-value < 0.200)

| IPA Run | Pathway | BH q-value |
| --- | --- | --- |
| 1 | | |
|  | Fcë_ Receptor-mediated Phagocytosis in Macrophages and Monocytes | 0.110 |
|  | Sperm Motility | 0.110 |
|  | PI3K Signaling in B Lymphocytes | 0.110 |
|  | Fc Epsilon RI Signaling | 0.121 |
|  | Complement System | 0.137 |
|  | IL-8 Signaling | 0.137 |
| 2 | | |
|  | RAR Activation | 0.153 |
| 3 | | |
|  | Glioma Invasiveness Signaling | 0.198 |
|  | Anandamide Degradation | 0.198 |
|  | FcγRIIB Signaling in B Lymphocytes | 0.198 |
|  | G Beta Gamma Signaling | 0.198 |
|  | Axonal Guidance Signaling | 0.198 |
|  | Actin Nucleation by ARP-WASP Complex | 0.198 |
|  | ILK Signaling | 0.198 |
|  | Integrin Signaling | 0.198 |
|  | Fatty Acid β-oxidation I | 0.198 |
|  | CXCR4 Signaling | 0.198 |
|  | Telomerase Signaling | 0.198 |
|  | Thrombin Signaling | 0.198 |
|  | GABA Receptor Signaling | 0.198 |
|  | N-acetylglucosamine Degradation II | 0.198 |
|  | Macropinocytosis Signaling | 0.198 |
|  | Thrombopoietin Signaling | 0.198 |
|  | CREB Signaling in Neurons | 0.198 |
|  | Type II Diabetes Mellitus Signaling | 0.198 |
|  | Prolactin Signaling | 0.198 |
| 4 | | |
|  | Integrin Signaling | 0.018 |
|  | Actin Cytoskeleton Signaling | 0.119 |
|  | TREM1 Signaling | 0.154 |
|  | Regulation of Actin-based Motility by Rho | 0.154 |
|  | Fcγ Receptor-mediated Phagocytosis in Macrophages and Monocytes | 0.154 |
|  | Glioblastoma Multiforme Signaling | 0.154 |
|  | PI3K Signaling in B Lymphocytes | 0.156 |
| 5 | | |
|  | Regulation of Actin-based Motility by Rho | 0.031 |
|  | Actin Cytoskeleton Signaling | 0.095 |
|  | Actin Nucleation by ARP-WASP Complex | 0.098 |
|  | Thrombin Signaling | 0.098 |
|  | Glioblastoma Multiforme Signaling | 0.134 |
|  | NF-κB Signaling | 0.134 |
|  | Caveolar-mediated Endocytosis Signaling | 0.168 |
|  | Signaling by Rho Family GTPases | 0.168 |
|  | HER-2 Signaling in Breast Cancer | 0.168 |
|  | GDP-glucose Biosynthesis | 0.168 |
|  | RhoA Signaling | 0.168 |
|  | Integrin Signaling | 0.169 |
|  | Epithelial Adherens Junction Signaling | 0.173 |
|  | Glucose and Glucose-1-phosphate Degradation | 0.173 |
|  | Sumoylation Pathway | 0.188 |
| 6 | | |
|  | T Cell Receptor Signaling | 0.013 |
|  | Role of NFAT in Regulation of the Immune Response | 0.023 |
|  | iCOS-iCOSL Signaling in T Helper Cells | 0.046 |
|  | Germ Cell-Sertoli Cell Junction Signaling | 0.046 |
|  | Gαq Signaling | 0.051 |
|  | Glioma Invasiveness Signaling | 0.055 |
|  | GDNF Family Ligand-Receptor Interactions | 0.076 |
|  | Angiopoietin Signaling | 0.076 |
|  | IL-8 Signaling | 0.076 |
|  | Regulation of IL-2 Expression in Activated and Anergic T Lymphocytes | 0.076 |
|  | CXCR4 Signaling | 0.076 |
|  | Thrombin Signaling | 0.076 |
|  | CD28 Signaling in T Helper Cells | 0.076 |
|  | Semaphorin Signaling in Neurons | 0.076 |
|  | HMGB1 Signaling | 0.076 |
|  | Tec Kinase Signaling | 0.078 |
|  | Colorectal Cancer Metastasis Signaling | 0.079 |
|  | Pancreatic Adenocarcinoma Signaling | 0.079 |
|  | Molecular Mechanisms of Cancer | 0.079 |
|  | Relaxin Signaling | 0.081 |
|  | Chronic Myeloid Leukemia Signaling | 0.087 |
|  | Phospholipase C Signaling | 0.106 |
|  | Th2 Pathway | 0.130 |
|  | Assembly of RNA Polymerase I Complex | 0.137 |
|  | CTLA4 Signaling in Cytotoxic T Lymphocytes | 0.139 |
|  | Th1 Pathway | 0.139 |
|  | Lymphotoxin β Receptor Signaling | 0.147 |
|  | Cardiac Hypertrophy Signaling | 0.151 |
|  | Role of IL-17A in Arthritis | 0.160 |
|  | 3-phosphoinositide Biosynthesis | 0.163 |
|  | NF-κB Activation by Viruses | 0.163 |
|  | Antiproliferative Role of TOB in T Cell Signaling | 0.168 |
|  | Cell Cycle Control of Chromosomal Replication | 0.180 |
|  | Th1 and Th2 Activation Pathway | 0.182 |
| 7 | | |
|  | Aryl Hydrocarbon Receptor Signaling | 0.046 |
|  | T Helper Cell Differentiation | 0.046 |
|  | Acute Phase Response Signaling | 0.133 |
|  | Role of JAK family kinases in IL-6-type Cytokine Signaling | 0.143 |
|  | Pyridoxal 5'-phosphate Salvage Pathway | 0.143 |
|  | PI3K Signaling in B Lymphocytes | 0.143 |
| 8 | | |
|  | T Cell Receptor Signaling | 0.012 |
| 9 | | |
|  | Osteoarthritis Pathway | 0.087 |
| 10 | No pathways significant for BH q-value < 0.20 | |
